# The Emotional Stroop Effect Is Modulated by the Biological Salience and Motivational Intensity Inherent in Stimuli

**DOI:** 10.3389/fpsyg.2019.03023

**Published:** 2020-01-21

**Authors:** Sixiang Quan, Zhenhong Wang, Ya Liu

**Affiliations:** ^1^School of Psychology, Shaanxi Normal University, Xi’an, China; ^2^School of Educational Science, Shaanxi Xueqian Normal University, Xi’an, China; ^3^Department of Psychology, Chongqing Normal University, Chongqing, China

**Keywords:** emotional Stroop effect, negative stimuli, positive stimuli, biological salience, motivational intensity

## Abstract

Prior research has found significant emotional Stroop effects for negative stimuli, but the results have been inconsistent for positive stimuli. Combining an evolutionary perspective of emotion with the motivational dimensional model of affect, we speculated that the emotional Stroop effect of a stimulus may be influenced by the biological salience and inherent motivational intensity of the stimulus. In the present study, we examined this issue with two experiments. The results indicated that both low- and high-withdrawal-motivation negative stimuli produced a robust emotional Stroop effect; however, the high-withdrawal-motivation negative stimuli produced a stronger emotional Stroop effect than the low-withdrawal-motivation negative stimuli. Regarding positive stimuli, only the high-approach-motivated positive stimuli produced the emotional Stroop effect, unlike the low-approach-motivation positive stimuli. These findings suggest that the emotional Stroop effect is modulated by the biological salience of stimuli and by the motivational intensity inherent in the stimuli. Biological salience and motivational intensity play an additive effect in the emotional Stroop effect.

## Introduction

The emotional Stroop effect refers to the phenomenon that the emotional information of stimuli will delay the reaction of participants when they are asked to respond to the non-emotional information in a task (Williams et al., 1996; Algom et al., 2004). Since the discovery of the emotional Stroop effect, researchers have examined this effect with different task stimuli, such as emotionally charged words (Algom et al., 2004; Chajut et al., 2010; Ben-Haim et al., 2014; Caparos and Blanchette, 2014), emotional pictures (Hester et al., 2006), facial expressions (Lee et al., 2009), and even sensitive stimuli for clinical populations (Andersson et al., 2006).

Many studies have demonstrated that the emotional Stroop effect of negative stimuli is significant (Williams et al., 1996; Algom et al., 2004; Frings and Wühr, 2012). It is believed that the negative stimuli, regardless of whether the stimuli are emotional words, emotional pictures or other types of stimuli, produce a robust emotional Stroop effect because the negative stimuli provide threat or alert information that is vital to survival (Fox et al., 2001; Schimmack and Derryberry, 2005; Wyble et al., 2008). Researchers have suggested that a dedicated system automatically captures or grasps negative threatening stimuli and prioritizes the processing of such stimuli (Algom et al., 2004; Brosch et al., 2011), and this automatic response to threatening stimuli shares many features with “automatic vigilance” (Pratto, 1994; Wentura et al., 2000). Thereby, our ongoing processing of target tasks can be interrupted by the threat information of negative stimuli, which automatically captures and occupies our attention and has priority processing (Algom et al., 2004; Reynolds and Langerak, 2015; Yamaguchi and Harwood, 2015). Therefore, negative threatening stimuli will always induce an emotional Stroop effect.

Regarding positive stimuli, the findings of studies on the emotional Stroop effect have been inconsistent. Some studies found the emotional Stroop effect of positive stimuli was significant (e.g., Martin et al., 1991; Constantine et al., 2001), but some other studies have not found this effect (e.g., McKenna and Sharma, 1995; White, 1996; Bertels et al., 2011). However, from an evolutionary perspective of emotion, positive stimuli such as infants and appetizing food or erotic stimuli are positive rewarding stimuli, and acquiring the sources of these stimuli is critical for an organism’s survival or reproduction. Therefore, similar to negative stimuli, these kinds of positive rewarding stimuli may capture attention automatically and have priority processing because such stimuli are particularly important for the acquisition of food, reproduction and social attachment (Gable and Harmon-Jones, 2010; Pool et al., 2016). Thus, these kinds of positive rewarding stimuli are highly biologically salient stimuli (Lykins et al., 2006; Brosch et al., 2007; Nummenmaa et al., 2011) and thus may produce an emotional Stroop effect. Different from positive stimuli such as appetizing food, infants and erotic stimuli, other positive stimuli such as scenery, flowers and sports pictures, which indicate a safe, comfortable environment in which conditions are better than necessary, suggest a low urgency to act and lead to relaxation (Fredrickson and Branigan, 2005; Gable and Harmon-Jones, 2010). That is, those positive stimuli that suggest a safe, comfortable environment in which conditions are better than necessary are stimuli with low biological salience and may not induce the emotional Stroop effect. However, no study to date has examined this issue.

Furthermore, negative and positive stimuli can be conceptualized as being diametrically opposed to each other in terms of valence and action tendency (Pool et al., 2016). Negative stimuli have a negative value, eliciting avoidance or withdrawal behaviors, and positive stimuli have a positive value, eliciting approach behaviors (Schultz, 2004; Berridge and Kringelbach, 2008; Chajut et al., 2010). According to the motivational dimensional model of affect proposed by Gable and Harmon-Jones (2010), negative stimuli can be divided into low- and high-withdrawal motivation negative stimuli, and positive stimuli can be divided into low- and high-approach motivation positive stimuli in terms of the motivational intensity inherent in emotional stimuli. Therefore, a negative stimulus such as a poisonous snake is a high-threat or high-withdrawal-motivation negative stimulus, and a negative stimulus such as pollution is a low-threat or low-withdrawal-motivation negative stimulus; conversely, positive stimuli such as appetizing food are high-rewarding or high-approach-motivation positive stimuli, and positive stimuli such as scenery are low-rewarding or low-approach-motivation positive stimuli. The motivational dimensional model of affect advocates that the effects of emotion on cognitive processing are modulated by the motivational intensity inherent in an emotional stimulus, and a large number of studies have provided evidence for this conclusion (Fredrickson and Branigan, 2005; Gable and Harmon-Jones, 2010; Liu and Wang, 2014). In the view of the motivational dimensional model of affect, high-motivation emotional stimuli, regardless of whether they are negative or positive stimuli, occupy more attentional resources or narrow our attention scope, while low-motivation emotional stimuli, regardless of whether they are negative or positive stimuli, occupy less attentional resources or broaden our attention scope (Gable and Harmon-Jones, 2008; Harmon-Jones et al., 2013). Therefore, emotional stimuli with high motivational intensity regardless of whether they are negative or positive stimuli might induce an emotional Stroop effect, while emotional stimuli with low motivational intensity regardless of whether they are negative or positive stimuli may not induce the same effect. However, little is known about whether the emotional Stroop effect could be modulated by the motivational intensity inherent in emotional stimuli.

In summary, the present study was conducted to examine whether the emotional Stroop effect is modulated by the biological salience of emotional stimuli and by the motivational intensity inherent in these stimuli. To examine this issue, two experiments were conducted in the present study. Considering the evolutionary perspective of emotion and the motivational dimensional model of affect, both high- and low-withdrawal-motivation negative stimuli may produce an emotional Stroop effect because an affective evaluation lends such stimuli a high survival value and the stimuli are thus more biologically salient. High-withdrawal-motivation negative stimuli produce stronger emotional Stroop effects than low-withdrawal-motivation negative stimuli. Regarding positive stimuli, high-approach-motivation positive stimuli, such as appetizing food, are highly biologically salient, suggesting that such stimuli may produce an emotional Stroop effect. Low-approach-motivation positive stimuli that suggest a safe, comfortable environment are stimuli with low biological salience and, therefore, may not produce an emotional Stroop effect.

## Experiment 1

To examine how both low- and high-withdrawal-motivation negative stimuli produce a robust emotional Stroop effect and whether high-withdrawal-motivation negative stimuli produce a stronger emotional Stroop effect than low-withdrawal-motivation negative stimuli, both high- and low-withdrawal-motivation negative pictures were used in experiment 1.

### Method

#### Participants

*A priori* sample size estimation with a large effect size and 95% statistical power was conducted by G^∗^Power to determine the sample size required for this study (Cohen et al., 2003). As the minimum number of participants required for the repeated-measures ANOVA with the large effect size (*f*^2^ = 0.40) and 95% statistical power was 18. Therefore, we recruited 47 undergraduate students (between 18 and 24 years old) with normal or corrected-to-normal vision participated in this experiment. None of the participants were color blind or had color weakness, and the participants could correctly distinguish all the colors used in our experiments. All of the participants were unaware of the purpose of this experiment. Each participant signed an informed consent form prior to the experiment. One participant decided not to continue with the experiment because of fear. The data from two other participants were excluded from the analysis because the accuracy of the data was lower than 90%. Thus, a total of 44 effective participants were included with a mean age of 21.65 years (*SD* = 1.44). After completing the experiment, the participants watched 5-min comedy clips of “Lost on Journey” to improve their mood. Each participant was fully debriefed as to the purpose of this experiment and obtained 30 RMB (4.22 $) as a reward.

#### Experiment Materials

The stimuli were chosen from the international affective picture system (IAPS) (Lang et al., 2005), the Chinese affective picture system (CAPS) (Lu et al., 2005), and the internet. We selected 112 emotional pictures in total. There were 32 sad and pollution pictures as the low-withdrawal-motivation stimuli^1^, 32 mutilation, snake and threat pictures as high-withdrawal-motivation stimuli^2^, and 32 household pictures and neutral daily life scenes pictures as neutral stimuli. The 16 remaining neutral stimuli were used as test stimuli in the practice session.^3^ We roughly matched the perceptual features, including color, brightness, and visual complexity, of the three kinds of pictures. The size of all the pictures was set to 192 × 144 pixels. We used Photoshop software to add 10-pixel green and red frames to each picture. Each picture appeared twice in the experiment: once with a red frame and once with a green frame.

#### Procedure

There were three experimental blocks (blocks with low-withdrawal-motivation, neutral, and high-withdrawal-motivation stimuli) with each block consisting of 64 trials. The presentation order of these stimuli was random and different for each participant. There was a 1-min rest break between each experimental block.

**FIGURE 1:**
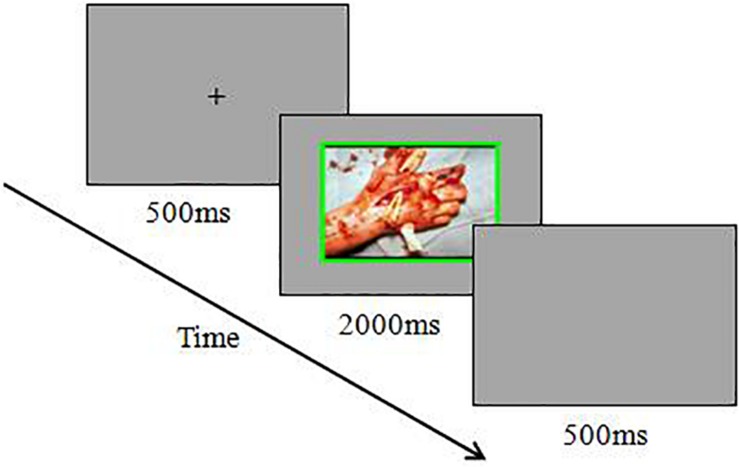
Schematic diagram for the emotional Stroop task in high-withdrawal-motivation negative stimuli trial.

Each trial started with a fixation cross for 500 ms. Afterward, the emotional pictures appeared and remained at the center of the screen for 2000 ms or until the participants gave a response. Participants were instructed to press the “S” key on the keyboard with the left index finger if the frame of a picture was red and to press the “K” key on the keyboard with the right index finger if the frame of a picture was green. They were asked only to judge the frame color and to ignore the content and color of the pictures. The participants were asked to respond as quickly and accurately as possible. The next trial began after a blank screen lasting 500 ms. To familiarize the participants with the experimental procedure, each participant was instructed to complete a practice block with 32 trials before the experimental block. After completing each experimental block, the participants were instructed to rate the pleasure (1 = extremely unpleasant, 9 = extremely pleasant), arousal (1 = extremely calm, 9 = extremely exciting), and motivational intensity (1 = especially want to withdrawal, 9 = especially want to approach) of the pictures that they just saw. An example of a trial for this experiment is shown in Figure 1.

### Results

Table 1 presents the means and standard deviations of the valence, arousal and motivational intensity of the three types of negative pictures. A repeated-measures ANOVA showed that there were significant effects on the valence, *F*(2,86) = 74.12, *p* < 0.001, ${\text{η}}_{P}^{2}$ = 0.63. *Post hoc* comparisons demonstrated that the pleasure ratings of both the high-withdrawal-motivation (*t*(43) = 13.92, *p* < 0.001, *d* = 2.56, 95% CI [1.91, 3.21]) and low-withdrawal-motivation negative pictures (*t*(43) = 6.08, *p* < 0.001, *d* = 1.43, 95% CI [0.87, 1.98]) were significantly lower than those of the neutral pictures. And the difference between high- and low-withdrawal-motivation negative pictures was also significant (*t*(43) = 5.42, *p* < 0.001, *d* = 1.13, 95% CI [0.65, 1.60]). On the arousal ratings, results suggested a significant effect for picture type, *F*(2,86) = 22.61, *p* < 0.001, ${\text{η}}_{P}^{2}$ = 0.35. The high-withdrawal-motivation (*t*(43) = 5.96, *p* < 0.001, *d* = 1.21, 95% CI [0.73, 1.68]) and low-withdrawal-motivation pictures (*t*(43) = 5.62, *p* < 0.001, *d* = 1.07, 95% CI [0.63, 1.50]) had significantly higher ratings than the neutral pictures. And the difference between high- and low-withdrawal-motivation negative pictures was not significant (*t*(43) = 1.91, *p* = 0.06, *d* = 0.38, 95% CI [−0.02, 0.78]). On the motivational intensity ratings, the effect of picture type of also significant, *F*(2,86) = 33.98, *p* < 0.001, ${\text{η}}_{P}^{2}$ = 0.44, with high-withdrawal-motivation (*t*(43) = 7.66, *p* < 0.01, *d* = 1.74, 95% CI [1.15, 2.30]) and low-withdrawal-motivation (*t*(43) = 5.53, *p* < 0.01, *d* = 1.21, 95% CI [0.71, 1.70]) pictures being higher than neutral pictures, and high-withdrawal-motivation pictures being higher than low-withdrawal-motivation pictures (*t*(43) = 3.46, *p* = 0.001, *d* = 0.81, 95% CI [0.32, 1.31]).

**TABLE 1:** Means and standard deviations of the negative pictures in valence, arousal, and motivational intensity ratings.

**Block**	**Valence**	**Arousal**	**Motivational intensity**
High-withdrawal	2.32 (1.38)	5.80 (2.11)	2.50 (1.85)
Low-withdrawal	3.82 (1.28)	5.11 (1.39)	3.77 (1.18)
Neutral	5.55 (1.13)	3.59 (1.47)	5.27 (1.30)

*Smaller rating number represents higher withdrawal-motivated intensity.*

**FIGURE 2:**
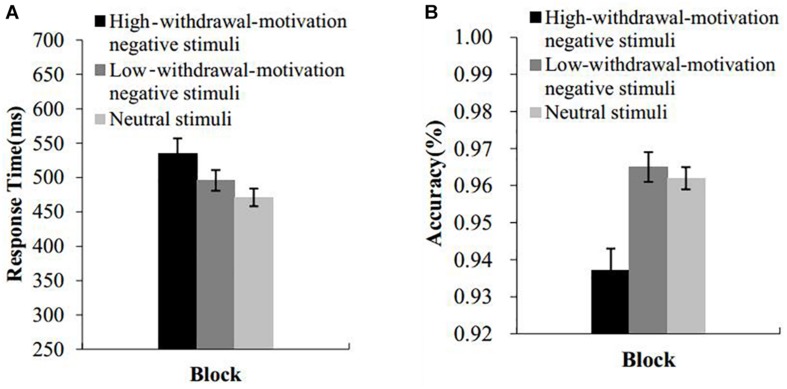
Results of experiment 1. RTs **(A)** and accuracy **(B)** of the emotional Stroop effect task as a function of block. Error bars represent standard errors.

Figure 2 presents the mean response times and accuracy for naming the color. Incorrect responses and responses with the response time more than three standard deviations from the mean were excluded. A repeated-measures ANOVA with picture type as a within-subject factor was conducted on the RT and accuracy data. The results indicated that the main effect of picture type on the response time was significant, *F*(2,86) = 14.71, *p* < 0.001, ${\text{η}}_{P}^{2}$ = 0.26. *Post hoc* comparisons revealed that response times were significantly longer under the block of high-withdrawal-motivation pictures than under the block of the low-withdrawal-motivation pictures (*t*(43) = 2.96, *p* < 0.01, *d* = 0.31, 95% CI [0.09, 0.53]) and the neutral pictures (*t*(43) = 4.73, *p* < 0.001, *d* = 0.54, 95% CI [0.29, 0.79]). There was also a significant difference in the response time between the low-withdrawal-motivation picture block and the neutral picture block (*t*(43) = 3.04, *p* < 0.01, *d* = 0.27, 95% CI [0.08, 0.45]).

The main effect of picture type on accuracy was significant, *F*(2,86) = 19.10, *p* < 0.001, ${\text{η}}_{P}^{2}$ = 0.31. *Post hoc* comparisons revealed that the accuracy under the block of the high-withdrawal-motivation pictures was significantly lower than that under the block of the low-withdrawal-motivation pictures (*t*(43) = 4.93, *p* < 0.001, *d* = 0.80, 95% CI [0.44, 1.16]) and the neutral pictures (*t*(43) = 4.81, *p* < 0.001, *d* = 0.82, 95% CI [0.44, 1.19]). There was no difference in accuracy between the block of low-withdrawal-motivation pictures and neutral pictures (*t*(43) = 0.59, *p* = 0.56, *d* = 0.09, 95% CI [−0.22, 0.40]).

### Discussion

The results of experiment 1 revealed that, compared with the neutral stimuli, both the high- and low-withdrawal-motivation negative stimuli produced significant interference. Existing studies have found that negative stimuli produce a robust emotional Stroop effect, and our findings are consistent with those prior findings (McKenna and Sharma, 1995; White, 1996). The biological salience of these stimuli is high. Furthermore, this study also found that the high-withdrawal-motivation negative stimuli produced stronger emotional Stroop effects than the low-withdrawal-motivation negative stimuli. Therefore, we believe that, although negative stimuli can produce the robust emotional Stroop effect, this effect may also be modulated by the motivational intensity inherent in the negative stimuli.

## Experiment 2

To examine whether high-approach-motivation positive stimuli produce the emotional Stroop effect but low-approach-motivation positive stimuli do not, high- and low-approach-motivation positive pictures were used in experiment 2.

### Method

#### Participants

Same as experiment 1, the number of participants required in this experiment were 18. Therefore, we recruited thirty undergraduate students with normal or corrected-to-normal vision (between 19 and 24 years old) participated in this experiment. None of the participants were color blind or had color weakness. The participants could correctly distinguish all the colors used in our experiments. All of them were unaware of the purpose of this experiment. Each participant signed an informed consent form prior to the experiment. After completing the experiment, the participants obtained 30 RMB (4.22 $) as a reward. One participant decided not to continue participation in the experiment for a personal reason. The data from two other participants were excluded from the analysis because the accuracy was lower than ninety percent. Thus, there were 27 effective participants with a mean age of 21.74 years (*SD* = 1.53).

#### Experiment Materials

The stimuli were chosen from the IAPS (Lang et al., 2005), the CAPS (Lu et al., 2005), and the internet. We selected 112 emotional pictures in total. There were 32 sports/adventure, scenery and happy pictures used as low-approach-motivation stimuli^4^, 32 appetizing dessert, infant, and erotic pictures used as high-approach-motivation stimuli^5^, and 32 household pictures and neutral daily life scenes pictures used as neutral stimuli (Briggs and Martin, 2009). The 16 remaining neutral stimuli were used in the practice session.^6^ We roughly matched the perceptual features, including color, brightness and visual complexity, of the three kinds of pictures. The size of all the pictures was set to 192 × 144 pixels. We used Photoshop software to add 10-pixel green and red frames at the margins of each picture. Each picture appeared twice in the experiment: once with a red frame and once with a green frame.

#### Procedure

**FIGURE 3:**
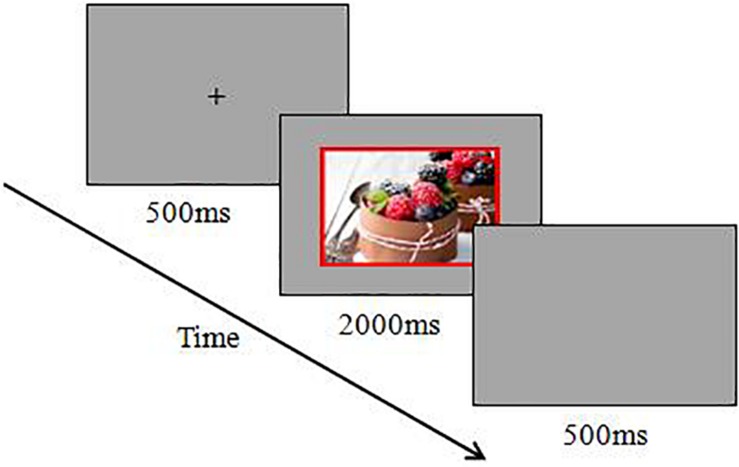
Schematic diagram for the emotional Stroop task in high-approach-motivation positive stimuli trial.

The procedure of experiment 2 was identical to that of experiment 1. An example of a trial for this experiment is shown in Figure 3.

### Results

Table 2 presents the means and standard deviations of the valence, arousal, and motivational intensity for the three types of positive stimuli. A repeated-measures ANOVA showed that there were significant effects on the valence, *F*(2,52) = 11.85, *p* < 0.001, ${\text{η}}_{P}^{2}$ = 0.31. *Post hoc* comparisons demonstrated that the pleasure ratings of both the high-approach-motivation (*t*(26) = 8.23, *p* < 0.001, *d* = 1.46, 95% CI [0.93, 2.00]) and low-approach-motivation pictures (*t*(26) = 2.65, *p* = 0.01, *d* = 0.81, 95% CI [0.17, 1.45]) were significantly higher than those of the neutral pictures. And the difference between high- and low-approach-motivation pictures was not significant (*t*(26) = 1.52, *p* = 0.14, *d* = 0.45, 95% CI [−0.15, 1.05]). On the arousal ratings, results suggested a significant effect for picture type, *F*(2,52) = 10.33, *p* < 0.001, ${\text{η}}_{P}^{2}$ = 0.28. The high-approach-motivation (*t*(26) = 4.27, *p* < 0.001, *d* = 1.21, 95% CI [0.56, 1.85]) and low-approach-motivation pictures (*t*(26) = 3.59, *p* = 0.001, *d* = 0.77, 95% CI [0.29, 1.23]) had significantly higher ratings than the neutral pictures. And the difference between high- and low-approach-motivation pictures was not significant (*t*(26) = 1.28, *p* = 0.21, *d* = 0.39, 95% CI [−0.22, 0.98]). On the motivational intensity ratings, the effect of picture type was also significant, *F*(2,52) = 11.71, *p* < 0.001, ${\text{η}}_{P}^{2}$ = 0.31. High-approach-motivation pictures had higher motivational intensity ratings than low-approach-motivation pictures (*t*(26) = 2.39, *p* = 0.02, *d* = 0.66, 95% CI [0.09, 1.22]) and neutral pictures (*t*(26) = 5.60, *p* < 0.01, *d* = 1.40, 95% CI [0.77, 2.01]), and the difference between low-approach-motivation pictures and neutral pictures was also significant (*t*(26) = 2.14, *p* = 0.04, *d* = 0.54, 95% CI [0.02, 1.04]).

**TABLE 2:** Means and standard deviations of the positive pictures in valence, arousal, and motivational intensity ratings.

**Block**	**Valence**	**Arousal**	**Motivational intensity**
High-approach	7.19 (1.44)	5.78 (1.48)	6.89 (1.55)
Low-approach	6.44 (1.81)	5.15 (1.77)	5.74 (1.91)
Neutral	5.15 (1.35)	3.78 (1.81)	4.85 (1.35)

**FIGURE 4:**
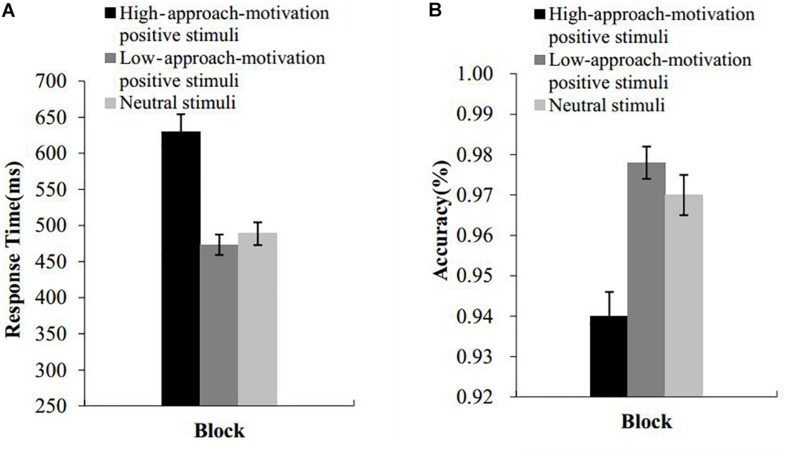
Results of experiment 2. RTs **(A)** and accuracy **(B)** of the emotional Stroop effect task as a function of block. Error bars represent standard errors.

The means and standard deviations of the response times and accuracy under the three blocks of high-approach-motivation stimuli, low-approach-motivation stimuli, and neutral stimuli are presented in Figure 4. Incorrect responses and response with the responses time more than three standard deviations from the mean were excluded. A repeated-measures ANOVA with picture type as a within-subject factor was conducted on the RT and accuracy data. The results indicated that the main effect of the picture type on the response time was significant, *F*(2,52) = 47.27, *p* < 0.001, ${\text{η}}_{P}^{2}$ = 0.65. *Post hoc* comparisons revealed that the response time was significantly longer under the high-approach-motivation picture block than under the low-approach-motivation picture block (*t*(26) = 8.58, *p* < 0.001, *d* = 1.47, 95% CI [0.95, 1.99]) and the neutral picture block (*t*(26) = 6.65, *p* < 0.001, *d* = 1.29, 95% CI [0.77, 1.80]). There was no difference between the low-approach-motivation picture block and the neutral picture block (*t*(26) = 1.22, *p* = 0.24, *d* = 0.20, 95% CI [−0.13, 0.52]).

The main effect of the picture type on accuracy was significant, *F*(2,52) = 23.68, *p* < 0.001, ${\text{η}}_{P}^{2}$ = 0.48. *Post hoc* comparisons revealed that the accuracy under the high-approach-motivation picture block was significantly lower than that under the low-approach-motivation pictures block (*t*(26) = 5.77, *p* < 0.001, *d* = 1.38, 95% CI [0.77, 1.97]) and neutral pictures block (*t*(26) = 4.92, *p* < 0.001, *d* = 1.03, 95% CI [0.53, 1.51]). There was no difference between the low-approach-motivation picture block and the neutral picture block (*t*(26) = 1.68, *p* = 0.11, *d* = 0.38, 95% CI [−0.06, 0.67]).

### Discussion

Experiment 2 revealed that the high-approach-motivation positive stimuli produced a significant emotional Stroop effect, while the low-approach-motivation positive stimuli caused no effect. These results supported our hypothesis that high-approach-motivation positive stimuli could produce the emotional Stroop effect, whereas the low-approach-motivation positive stimuli could not.

## General Discussion

Results of current study indicate that both the high- and low-withdrawal-motivation negative stimuli produced a robust emotional Stroop effect, and the high-withdrawal-motivation negative stimuli produced a stronger emotional Stroop effect than the low-withdrawal-motivation negative stimuli. Regarding positive stimuli, only the high-approach-motivation positive stimuli produced an emotional Stroop effect, while the low-approach-motivation positive stimuli were ineffective at producing such an effect. These results may suggest that the emotional Stroop effect of stimuli is modulated by the biological salience and motivational intensity inherent in the stimuli.

Our study indicated that both high- and low-motivation negative stimuli produced a robust emotional Stroop effect. From the perspective of evolutionary theory, evolutionary pressure has led the nervous system (“negative brain”) to guarantee rapid and intense responses to negative stimuli (Carretié et al., 2009). Negative stimuli require processing and response resources to be more intensely and urgently mobilized. This urgency would have obvious adaptive and evolutionary advantages (Ekman, 1992; Ohman et al., 2000). Specifically, through evolution, when we encounter dangers or threat stimuli, which are referred to as high-withdrawal-motivation negative stimuli, our biological system acts as fast as possible to escape from the disadvantageous situations in order to survive or allow the continuation of the species. In other words, when we encounter unexpected threat stimuli while we are completing a task, the life-critical information must interrupt the current goal-directed processing. There is a balance between maintaining goal-directed processing and ensuring that the life-critical information interrupts the ongoing processing (Allport, 1989). The processing of this kind of stimuli is automatic and prioritized (Algom et al., 2004; Reynolds and Langerak, 2015; Yamaguchi and Harwood, 2015). The low-withdrawal-motivation negative stimuli indicate that there are unpleasant or dislike things around us. This state is also detrimental to our survival and well-being. Our attention should prioritize these stimuli in contrast to neutral stimuli. In a word, negative stimuli, regardless of whether the stimuli inherently high or low motivational intensity have, convey crucial information meaning that the organism should escape from the current environment or adjust actions. Thus, both high- and low-withdrawal motivation negative stimuli require attentional resources for the prioritized processing of these stimuli and thus delay the processing of a goal task (Augst et al., 2014). Once a stimulus captures one’s attention, we should then disengage from this stimulus and begin to process the next stimulus. Especially under the condition in which a stimulus contains a negative emotional task-irrelevant dimension, attentional disengagement from this dimension is difficult, hindering the processing of the task-relevant dimension of the next stimulus (Bertels et al., 2011). Taken together, due to the negative stimulus involved, both the high- and low-withdrawal-motivation negative stimuli were biologically salient, and the emotional Stroop effect was observed under both the high- and low-withdrawal-motivation negative stimuli blocks in the present study.

It seems that a failure to respond to negative information may lead to injury or even death, while a failure to respond to positive information may lead to missed opportunities (Hilgard et al., 2014). Generally, an insufficient response to positive stimuli seems to have less serious results. However, from an evolutionary point of view, focusing our attention on positive stimuli such as appetizing food or erotic stimuli is also critical to species survival. Positive stimuli such as appetizing food or erotic stimuli are more biologically salient stimuli and have high inherent approach motivation intensity. Both appetizing food and erotic stimuli are tightly connected to survival, choosing a mate and reproduction. An individual’s survival crucially depends not only on the ability to avoid disadvantageous situations but also on the ability to detect and acquire nourishment (Nummenmaa et al., 2011). Regardless of whether the attentional resources are sufficient, the positive stimuli with high motivational intensity that have high biological salience or convey survival information will be prioritized and processed automatically. Specifically, when we encounter things representing energy intake that can sustain our life, such as appetizing food, our brain is intrinsically prepared to process these stimuli. This brain mechanism is highly biologically plausible, as it makes the food-relevant information “pop out” from the multidimensional stimuli (Nummenmaa et al., 2011). Moreover, erotic and infant stimuli can also effectively capture our attention (Lykins et al., 2006; Brosch et al., 2007). Hence, when these kinds of information are task-irrelevant, such stimuli will produce interference in the current task. In addition, when we encounter low-approach-motivation positive stimuli, there is no emotional Stroop effect. In our daily life, the average resting mood of most people is quite positive rather than absolutely neutral (Gasper and Clore, 2002). This state made the participants have no significantly different reaction when they encountered the low-approach-motivation positive stimuli and the neutral stimuli. Low-approach-motivation positive stimuli are not very urgent for an organism’s survival. Our attention will still be allocated to the current task to complete goal-directed actions under this condition. Therefore, while high-approach-motivation positive stimuli produced a significant emotional Stroop effect, low-approach-motivation positive stimuli did not.

Moreover, although the present study found that both the high- and low-withdrawal-motivation negative stimuli produced a robust emotional Stroop effect, the high-withdrawal-motivation negative stimuli produced a stronger emotional Stroop effect than the low-withdrawal-motivation negative stimuli. This means that negative stimuli, regardless of having a high or low motivational intensity, produced a robust emotional Stroop effect; however, the negative stimuli with a higher inherent motivational intensity produced a stronger emotional Stroop effect. Therefore, this result suggests that the emotional Stroop effect of negative stimuli is also modulated by the inherent motivational intensity of such stimuli. Furthermore, as mentioned above, high-approach-motivation positive stimuli produced a significant emotional Stroop effect, but low-approach-motivation positive stimuli did not. That is, the biological salience and motivational intensity inherent in no matter negative or positive stimuli are additive factors which may contribute to the emotional Stroop effect. Therefore, the emotional Stroop effect might be modulated not only by the biological salience of emotional stimuli but also may be modulated by the inherent motivational intensity of such stimuli.

This study took an experiment approach to the emotional Stroop effect by investigating the biological salience and motivational intensity inherent in the stimuli. And these two factors have not received much attention in the emotional Stroop literatures. The findings provide the first evidence that biological salience and motivational intensity have additive effect for the emotional Stroop effect. These findings provide a new perspective and more comprehensive interpretation for this effect, and provide supports for the motivational dimensional model of affect.

Several limitations of our study should be considered. First, our study only adopted affective pictures as the stimuli, therefore, diverse stimuli should be used in future research to explore whether these findings can be generalized to emotional words or other conditioned emotional stimuli. Second, all the participants recruited in the present study were normal undergraduates, and thus, the results might not be generalized to clinical participants, such as people with anxiety, anorexia, post-traumatic stress disorder and so on.

## Conclusion

In conclusion, the present study found that both high-and low-withdrawal-motivation negative stimuli produced a robust emotional Stroop effect, and high-withdrawal-motivation negative stimuli produced a stronger emotional Stroop effect than low-withdrawal-motivation negative stimuli. High-approach-motivation positive stimuli produced an emotional Stroop effect, but low-approach-motivation positive stimuli did not. These findings provide the first evidence that the emotional Stroop effect is modulated by the biological salience of stimuli and by the motivational intensity inherent in the stimuli. Biological salience and motivational intensity play an additive effect in the emotional Stroop effect.

## Data Availability Statement

The datasets generated for this study are available on request to the corresponding author.

## Ethics Statement

The studies involving human participants were reviewed and approved by Institutional Review Board of the Psychology School of Shaanxi Normal University. The patients/participants provided their written informed consent to participate in this study.

## Author Contributions

ZW developed the study concept. SQ and YL contributed to the study design. SQ conducted the experiments and interpreted the data under the supervision of ZW. SQ drafted the manuscript. ZW and YL provided critical revisions. All authors approved the final version of the manuscript for submission.

## Conflict of Interest

The authors declare that the research was conducted in the absence of any commercial or financial relationships that could be construed as a potential conflict of interest.

## Footnotes

1 Sixteen pictures were chosen from IAPS. The picture numbers were 2141, 2205, 2278, 2385, 2399, 2455, 2700, 2722, 9001, 9220, 9280, 9290, 9342, 9421, 9560, and 9830. Four pictures were chosen from CAPS. The picture numbers were 3323, 3404, 3407, and 3429. Other twelve pictures which were similar to the pictures selected in IAPS or CAPS were chosen from the internet.

2 Twenty-seven pictures were chosen from IAPS. The picture numbers were 1019, 1050, 1052, 1080, 1090, 1114, 1200, 1205, 1300, 1301, 1321, 1525, 1721, 1726, 1932, 3015, 3051, 3060, 3064, 3068, 3071, 3080, 3100, 3150, 3170, 3181, and 9405. Three pictures were chosen from CAPS. The picture numbers were 3302, 3304, and 3308. Other two pictures which were similar to the pictures selected in IAPS or CAPS were chosen from the internet.

3 Twenty-seven pictures were chosen from IAPS. The picture numbers were 2190, 2200, 2210, 2214, 2215, 2383, 2393, 2394, 2441, 2630, 7002, 7009, 7010, 7020, 7034, 7035, 7042, 7052, 7055, 7150, 7175, 7190, 7217, 7233, 7550, 7705, and 7950. Two pictures were chosen from CAPS. The picture numbers were 2105 and 2055. Other nineteen stimuli which were similar to the pictures selected in IAPS or CAPS were chosen from the internet.

4 Twenty-two pictures were chosen from IAPS. The picture numbers were 2000, 2010, 2020, 5010, 5200, 5201, 5220, 5711, 8160, 8370, 8179, 8186, 8120, 8250, 8320, 8330, 8350, 8380, 8461, 8490, 8497, and 8540. Seven pictures were chosen from CAPS. The picture numbers were 1008, 1018, 1049, 1063, 1098, 1233, and 1244. Other three stimuli which were similar to the pictures selected in IAPS or CAPS were chosen from the internet.

5 Ten pictures were chosen from IAPS. The picture numbers were 2070, 4660, 4689, 4694, 4695, 4800, 7270, 7250, 7330, and 7430. Other twenty-two pictures which were similar to the pictures selected in IAPS were chosen from the internet.

6 The neutral pictures of experiment 1 and experiment 2 were the same.
